# Efficacy of a Pediatric Sleep Questionnaire for the Diagnosis of Obstructive Sleep Apnea in Children

**DOI:** 10.7759/cureus.12244

**Published:** 2020-12-23

**Authors:** Andrew M Ferry, Alex E Wright, Jason F Ohlstein, Kim Khoo, Harold S Pine

**Affiliations:** 1 Department of Otolaryngology, University of Texas Medical Branch, Galveston, USA

**Keywords:** polysomnography, screening, apnea-hypopnea index, respiratory disturbance index, cost, diagnosis, pediatrics

## Abstract

Background: Obstructive sleep apnea (OSA) is a source of significant morbidity in children. Polysomnography (PSG), the gold standard diagnostic tool for OSA, is often unavailable due to patient financial and geographic constraints. Our objective is to analyze the relationship between a patient's subjective complaints and the results from their PSG to determine the diagnostic value of the Pediatric Sleep Questionnaire (PSQ) for detecting OSA in children.

Methods: A retrospective chart review was conducted for pediatric patients with suspected OSA from March 2012 to January 2014. Preoperative PSQ scores were compared with the results from PSG in the form of Apnea-Hypopnea Index (AHI) and Respiratory Disturbance Index (RDI) values. AHI and RDI values ranging from 1 to 5 were classified as mild OSA while values ranging from 5 to 10 were classified as moderate OSA.

Results: A total of 161 patients were recruited for this study with 63 patients (39%) both completing the PSQ and undergoing PSG. Sensitivity of the positive questionnaire was higher in patients with AHI and RDI values indicative of moderate OSA (95% and 100% respectively) versus values indicative of mild OSA (83% and 86% respectively). Conversely, the positive predictive value of the positive questionnaire (n=49) was lower in patients with AHI and RDI values indicative of moderate OSA (39% and 46% respectively) versus values indicative of mild OSA (70% and 80% respectively).

Conclusion: The PSQ has high diagnostic value for screening patients with suspected OSA. We recommend the use of the PSQ in the primary care setting for children with suspected OSA.

## Introduction

Obstructive sleep apnea (OSA) affects between 1.2% to 5.7% of children and adolescents in the United States and is associated with significant morbidity [1]. OSA is characterized by episodic upper airway collapse with concomitant apnea or hypopnea throughout sleep [2-3]. These episodic collapses of the upper airway result in hypercarbia and desaturation of oxyhemoglobin resulting in episodes of sleep arousal [2-3]. The etiology of OSA in children is complex and multifactorial with adenotonsillar hypertrophy being the most common cause of obstruction [2-3]. Other risk factors associated with children developing OSA include obesity, prematurity, African American ancestry, abnormalities of the maxillofacial skeleton, and neuromuscular disorders [4-6]. Pediatric patients with OSA are at an increased risk for the development of cardiovascular complications secondary to chronic hypertension [7]. From a psychosocial standpoint, children with OSA exhibit higher rates of poor academic performance, depression, and emotional lability when compared to the general population [8].

Presently, polysomnography (PSG) in an attended, overnight setting is the gold standard diagnostic tool for detecting OSA in pediatric patients [9]. Despite its efficacy, PSG has several shortcomings that limit its overall utility. While PSG is highly sensitive and specific for OSA in children and adolescents, it has limited effectiveness for the diagnosis of pediatric OSA due to its high associated cost, inconvenient nature, and lack of availability in underserved regions [10].

These limitations have prompted healthcare specialists to develop diagnostic tools that are affordable and readily available. Chervin et al. designed the pediatric sleep questionnaire (PSQ) to predict sleep-related breathing disorders (SRBD). The study performed by Chervin et al., however, did not examine whether SRBD could be identified during an initial evaluation for a patient with suspected OSA [11]. To our knowledge, this is the first study to investigate the PSQ’s efficacy in pediatric patients with suspected OSA. Our objective is to assess the PSQ’s efficacy as a diagnostic modality for pediatric OSA by comparing the results of the questionnaire to the results of patients’ sleep studies.

## Materials and methods

We performed a retrospective chart review of patients under the age of 18 years who were evaluated by a pediatric otolaryngologist for suspected OSA from March 2012 to January 2014 at the University of Texas Medical Branch at Galveston (UTMB). During evaluation, patients’ legal guardians were asked to fill out the PSQ if patients had the following symptoms: daytime fatigue, morning headaches, snoring, sudden awakenings with a sensation of choking or gasping, irritability, trouble concentrating, or difficulty awakening in the morning (Table 1). We utilized the scoring system developed by Chervin et al. to ensure the results of this study would be generalizable to healthcare providers using the PSQ [11]. Answer choices for questions included “yes,” “no,” or “Don’t Know.” The “Yes” answer choices were scored as one point, the “no” answer choices were scored as 0 points, and the “Don’t Know” answers were excluded from questionnaire scoring. If an answer choice was not selected or if an ambiguous write-in answer was recorded, the question was scored as “Don’t Know.” Questionnaires were scored as: Score = (number of “yes” answers)/(total questions answered either "yes" or "no"). Questionnaire scores > 33% were deemed “positive.”

**Table 1:** Questions asked on the Pediatric Sleep Questionnaire (PSQ) [11].

Question
While sleeping, does your child…
…snore more than half the time?
…always snore?
…snore loudly?
…have “heavy” or loud breathing?
…have trouble breathing, or struggle to breathe?
Have you ever…
…seen your child stop breathing during the night?
Does your child…
…tend to breathe through the mouth during the day?
…have a dry mouth on waking up in the morning?
…occasionally wet the bed?
Does your child…
…wake up feeling unrefreshed in the morning?
…have a problem with sleepiness during the day?
Has a teacher or other supervisor commented that your child appears sleepy during the day?
Is it hard to wake your child up in the morning?
Does your child wake up with headaches in the morning?
Did your child stop growing at a normal rate at any time since birth?
Is your child overweight?
This child often…
…does not seem to listen when spoken to directly
…has difficulty organizing task and activities
…is easily distracted by extraneous stimuli
…fidgets with hands or feet or squirms in seat
…is ‘on the go’ or often acts as if ‘driven by a motor’
…interrupts or intrudes on others (e.g. butts into conversations or games)

The PSQ was available in both English and Spanish in order to prevent selection bias in our study. Patients with clinical suspicion for OSA underwent PSG at an UTMB or unaffiliated sleep center. PSG results, in the form of Apnea-Hypopnea Index (AHI) and Respiratory Disturbance Index (RDI) values, were used to determine if a patient had OSA. Patients with AHI and RDI values between 1 and 5 were classified as having mild OSA while patients with values between 5 and 10 were classified as having moderate OSA [12]. 

The sensitivity, specificity, positive predictive value (PPV), and negative predictive value (NPV) were calculated to assess the questionnaire’s ability to detect a pathologic result on PSG. Inter-rater reliability was calculated using Cohen’s kappa index (κ). PSQ and PSG results, clinic notes, lab results, and demographic information were gathered from the patient’s existing electronic medical records. No additional data was obtained from the patient directly. Patients were excluded from the study if either the sleep questionnaire or PSG were incomplete. Data including patient age upon questionnaire completion, answers to the questionnaire, questionnaire score, AHI score, and RDI score were recorded and subsequently analyzed in Microsoft Excel (Microsoft Inc., Redmond, WA, USA). This study was evaluated and approved by the UTMB Institutional Review Board.

## Results

A total of 161 patients were identified for inclusion in our study. Out of these patients, only 63 (39%) completed the PSQ and underwent PSG with 49 (30%) patients scoring above 33% on their sleep questionnaire.

The diagnostic properties of the PSQ are shown in Table 2. Out of the 49 patients with positive questionnaires, 34 (69%) patients had an AHI score greater than one while 19 (39%) patients had an AHI score greater than 5. We observed a mild correlation between AHI values greater than one and positive questionnaire scores (κ = 0.16). Similarly, we observed a mild correlation between AHI values greater than 5 and positive questionnaire scores (κ = 0.18). Out of the 49 patients with positive questionnaires, 33 patients had an RDI score greater than one while 19 patients had an RDI score greater than 5. We observed minimal correlation between RDI values greater than one and positive questionnaire scores (κ = 0.07). Conversely, we observed a mild correlation between RDI values greater than 5 and positive questionnaire scores (κ = 0.18).

**Table 2:** Diagnostic properties of the pediatric sleep questionnaire for the detection of pathologic polysomnography results. Abbreviations: PSG = Polysomnography, PPV = positive predictive value, NPV = negative predictive value, κ = kappa index, AHI = Apnea-Hypopnea Index, RDI = Respiratory Disturbance Index

	Number of patients with PSG result	Number of patients with positive questionnaire	Sensitivity	Specificity	PPV	NPV	κ
AHI							
>1	41	34	0.83	0.32	0.70	0.50	0.16
>5	20	19	0.95	0.30	0.39	0.93	0.18
RDI							
>1	38	33	0.86	0.20	0.80	0.29	0.07
>5	19	19	1.00	0.24	0.46	1.00	0.18

## Discussion

In this retrospective study we investigated the efficacy of the PSQ developed by Chervin et al. for the diagnosis of OSA in the pediatric population [11]. To our knowledge, this is the first study to investigate the efficacy of the PSQ in children with suspected OSA.

The PSQ had an observed high sensitivity for PSG values indicative of pediatric OSA. The observed sensitivity was high for detecting pathologic AHI values greater than 1 and 5 (0.83 and 0.95 respectively) and RDI values greater than 1 and 5 (0.86 and 1.00 respectively). The PSQ demonstrated higher sensitivity for patients with higher AHI and RDI values indicating that the questionnaire is more effective at detecting moderate to severe OSA than mild OSA. The observed specificity of the PSQ for the diagnosis of pediatric OSA was low at both AHI values greater than 1 and 5 (0.32 and 0.30 respectively) and RDI values greater than 1 and 5 (0.20 and 0.24 respectively). These findings suggest that the diagnostic properties of the PSQ make it ideal to be utilized as a diagnostic screening modality that is able to rule out OSA in pediatric patients with suspected OSA rather than as a confirmatory test [13]. 

While the sensitivity and specificity of the questionnaire were shown to be similar across AHI and RDI endpoints, the PPV and NPV were found to vary greatly. The PPV and NPV of the questionnaire for detecting AHI values greater than 1 were 0.70 and 0.50 respectively while the PPV and NPV of AHI values greater than 5 were 0.39 and 0.93 respectively. Similarly, the PPV and NPV of the questionnaire for detecting RDI values greater than 1 were 0.80 and 0.29 respectively while the PPV and NPV of RDI values greater than 5 were 0.46 and 1.00 respectively. These findings demonstrate that the PSQ detects a higher ratio of true positives to false positives when detecting AHI and RDI values indicative of mild to severe OSA as opposed to AHI and RDI values indicative of moderate to severe OSA. Furthermore, the PSQ detects a higher ratio of true negatives to false negatives at AHI values indicative of moderate to severe OSA than at AHI and RDI values indicative of mild to moderate OSA. We surmise that our observations are due to the higher prevalence of mild OSA than moderate and severe OSA in our study population as PPV and prevalence are positively correlated [14]. We believe this finding has generalizability as the literature suggests that the prevalence of patients with symptoms of mild OSA is much higher than national estimates that include moderate to severe OSA [15,16].

While the PSQ demonstrates efficacy as a screening tool for the diagnosis of OSA in the pediatric population, it is not without weaknesses. The primary weakness of the PSQ is how its scoring system calculates incomplete or ambiguous responses on the questionnaire. Any responses that were either incomplete, marked as “Don’t Know,” or were unintelligible were excluded from the total question count [11]. This exclusion allows the remaining “yes” and “no” responses to skew the final questionnaire score. This weakness in the PSQ is exaggerated by the inclusion of the DSM-IV diagnostic criteria for Attention-Deficit/Hyperactivity Disorder (ADHD) [11]. Despite evidence that the symptomatology of ADHD and pediatric OSA are similar, and in some cases related, they are relatively non-specific symptoms of OSA in children when compared to symptoms such as apneic episodes during sleep [17,18]. Additionally, the inattentive and hyperactive symptoms of ADHD are more noticeable in day-to-day life rendering them more susceptible to recall bias. Taking these factors into account, it can be surmised that the PSQ score is more heavily influenced by non-specific symptoms rather than specific symptoms. To avoid unnecessary evaluation for false-positive results, patients should be evaluated for ADHD before taking the PSQ.

**Figure 1:**
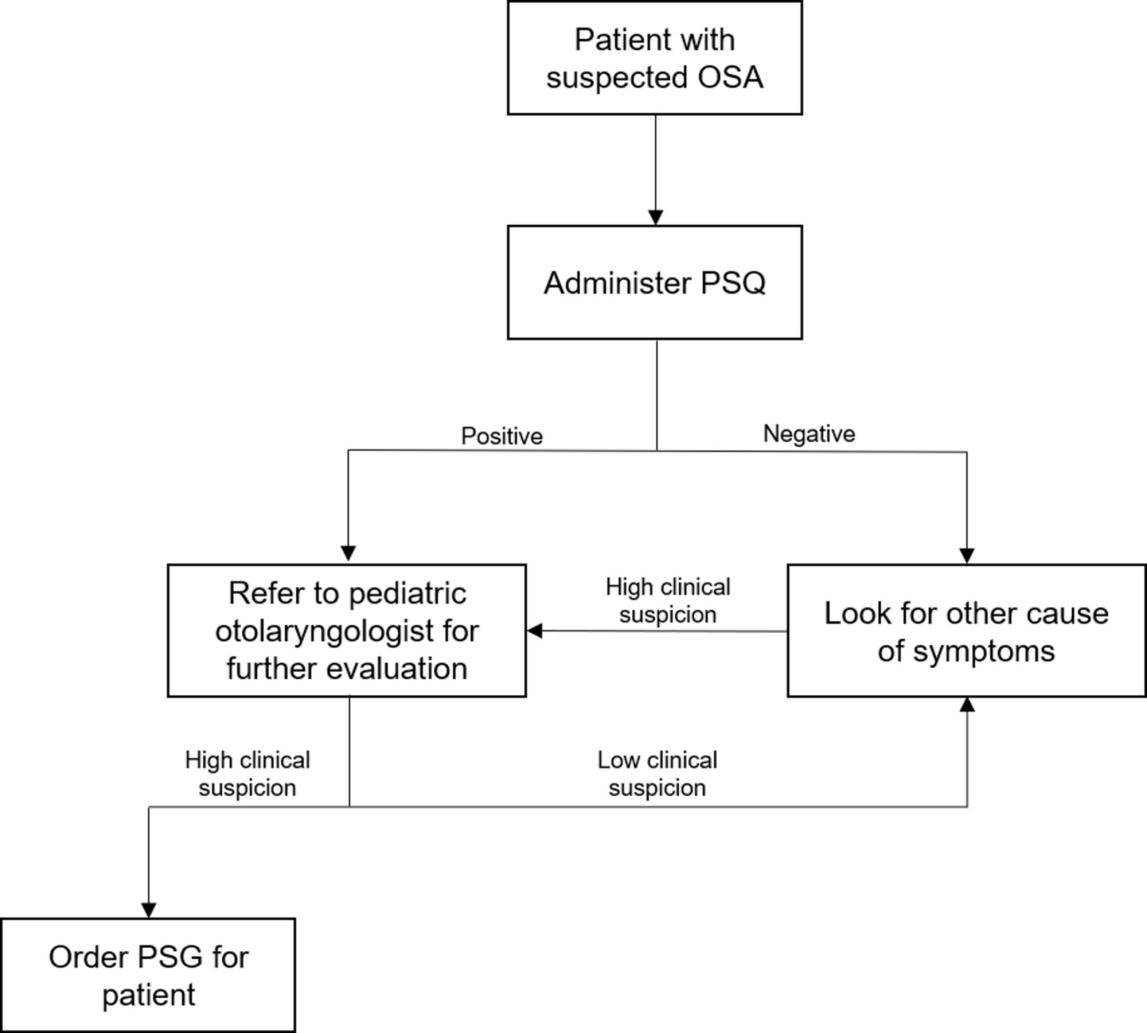
Management algorithm for suspected obstructive sleep apnea in a primary care setting. Abbreviations: OSA = Obstructive sleep apnea, PSQ = Pediatric sleep questionnaire, PSG = Polysomnography

Our findings suggest that the PSQ, despite its limitations, has utility as a screening tool for OSA in children and adolescents [19-22]. Our proposed algorithm for the diagnosis of OSA is illustrated in Figure 1. We recommend that the PSQ be administered in a primary care setting to reduce the number of unnecessary referrals to pediatric otolaryngologists. This, in turn, may result in fewer patients undergoing PSG and potentially shorten waiting times for patients who require PSG.

Our study has several limitations. First, the size of our study population increases the risk for skewing of results and type two error. Second, while all sleep centers measured patients’ AHI, several sleep centers did not measure patients’ RDI resulting in fewer data points. Third, there are no universally agreed upon AHI and RDI values for diagnosing or grading the severity of OSA in pediatric patients due to the significant anatomic heterogeneity exhibited across age groups [12,16]. Lastly, the results obtained from PSG have been demonstrated to lack consistency across sessions potentially limiting the quality of our data [23-27].

## Conclusions

The Pediatric Sleep Questionnaire demonstrated high sensitivity for Apnea-Hypopnea Index and Respiratory Disturbance Index values suggestive of obstructive sleep apnea in children and adolescents. The authors recommend that the Pediatric Sleep Questionnaire be administered in a primary care setting with goal of reducing inappropriate referrals to pediatric otolaryngologists. Further studies are needed to validate the Pediatric Sleep Questionnaire’s efficacy in the primary care setting and to assess its potential impact on the United States’ healthcare system.
